# HRV-Based Training for Improving VO_2max_ in Endurance Athletes. A Systematic Review with Meta-Analysis

**DOI:** 10.3390/ijerph17217999

**Published:** 2020-10-30

**Authors:** Antonio Granero-Gallegos, Alberto González-Quílez, Daniel Plews, María Carrasco-Poyatos

**Affiliations:** 1 Health and Public Administration Research Center, Department of Education, University of Almeria, 04120 Almeria, Spain; agranero@ual.es; 2 Department of Education, University of Almeria, 04120 Almeria, Spain; albertillo_gq@hotmail.com; 3 Sports Performance Research Institute, The Waikato University, Hamilton 3216, New Zealand; daniel.plews@aut.ac.nz; 4 School of Sport & Recreation, Auckland University of Technology, Auckland 92006, New Zealand

**Keywords:** performance, heart rate variability, high-level athletes, maximal oxygen uptake

## Abstract

This review aimed to synthesize evidence regarding interventions based on heart rate variability (HRV)-guided training for VO_2max_ improvements in endurance athletes and address the issues that impact this performance enhancement. The Cochrane Central Register of Controlled Trials (CENTRAL), MEDLINE, EMBASE, CINAHL Complete, the Web of Science Core Collection, Global Health, Current Contents Connect, and the SciELO citation index were searched. Inclusion criteria were: randomized controlled trials; studies with trained athletes enrolled in any regular endurance training; studies that recruited men, women, and both sexes combined; studies on endurance training controlled by HRV; studies that measured performance with VO_2max_. A random-effects meta-analysis calculating the effect size (ES) was used. Moderator analyses (according to the athlete’s level and gender) and metaregression (according to the number of participants in each group) were undertaken to examine differences in ES. HRV-guided training and control training enhanced the athletes’ VO_2max_ (*p* < 0.0001), but the ES for the HRV-guided training group was significantly higher (*p* < 0.0001; ES_HRVG-CG_ = 0.187). The amateur level and female subgroup reported better and significant results (*p* < 0.0001) for VO_2max_. HRV-guided training had a small (ES = 0.402) but positive effect on endurance athlete performance (VO_2max_), conditioned by the athlete’s level and sex.

## 1. Introduction

### 1.1. Description of the Condition

The key components in any training program are the volume (i.e., how much), intensity (i.e., how hard), and frequency (i.e., how often) of the exercise sessions, and the combination of these ‘training impulses’ determines the magnitude of adaptive responses that improve the physical condition of an athlete or increase fatigue [1]. Combining these key elements to optimize training in athletes for better performance represents a relevant area of research within exercise physiology and sports medicine [2]. It is recognized that a standard training program applied to a group of athletes can induce diverse responses in terms of performance and physiological adaptations [3,4]. Therefore, individualization is recognized as a training principle [1] as well as the need to adjust training stimuli to the psychophysical load capacity and individual tolerance of each athlete, if individual responses to training and recovery loads are intended for optimal performance [5]. The maximal oxygen uptake (VO_2max_) is considered one of the main indicators for measuring an athlete’s performance and cardiovascular adaptation to training loads [6]. The VO_2max_ is defined as the largest volume of oxygen that the body can capture, use, and transport during intense exercise [7] and is a determining factor of endurance performance [7,8]. As Vesterinen et al. [4,9] state, although some athletes show great endurance performance improvements after standardized group training (even up to 40% in VO_2max_), other athletes show no changes or benefits, and sometimes even show a decrease in endurance performance. In recent years, research has looked at whether heart rate variability (HRV)-guided training has positive effects on athletic performance, given that this type of training allows daily adjustment of the training and recovery stimuli, individually based on HRV records [4,5,10].

### 1.2. Description of the Intervention

HRV is an indicator that enables the noninvasive analysis of autonomic nervous system activity in both its sympathetic and parasympathetic branches [11]. This is relevant if we consider that an important component of the interindividual variability in physiological responses to training is related to the balance between the parasympathetic (PNS) and sympathetic (SNS) activity of the autonomic nervous system (ANS) [12]. According to Huang et al. [13], HRV is considered the variation in the time interval between two consecutive heartbeats and obtained by calculating the time interval between two consecutive R waves (i.e., RR interval fluctuation) in the electrocardiogram (ECG). Since the elapsed time between beats is not constant, high vagally related HRV values are associated with efficient ANS, promoting behavioral adaptation and cognitive flexibility during stress [14], while low HRV is indicative of an inefficient ANS, resulting in maladaptive responses to stress and perceived threats [13]. HRV analysis is considered a useful method for measuring the heart’s ability to adapt to endogenous and exogenous loads [15]; therefore, it can be used for the individual assessment of responses to training loads and recovery adaptation [4,16]. High HRV measurements indicate more parasympathetic than sympathetic activation, which is indicative of better recovery and preparedness for facing high-intensity training sessions [17]. HRV-guided training starts with a preparation period of about four weeks, which serves as a standardized data collection phase to obtain the baseline HRV values (e.g., LnrMSSD; the natural logarithm of the square root of the mean value of the sum of the squares of the differences between the adjacent RR intervals) and their normal range (upper and lower limits) for each athlete [9,18]. Once the normal range of HRV measurements has been established, the training prescribed (moderate- or high-intensity session) is based on this calculation, which is normally updated weekly [19]. Traditionally, the vagally related HRV index has been measured with ECG [20], and quantified by means of rMSSD [17]. Currently, the development and validation of new applications (i.e., smartphone applications: Kubios-HRV, Elite-HRV, Mobile Lab, or HRV4Training) facilitate daily HRV measurements and their quantification and, thus, the individual adaptation of training loads and recovery.

### 1.3. How the Intervention Might Work

Bellenger et al. [21], in a recent systematic review with meta-analysis, highlighted the need to use monitoring systems that accurately reflect the athletes’ adaptations to the training stimulus. Although there have been numerous research studies using the HRV measure to check wellness and training adaptation in athletes [22,23], these have not focused on performance improvement based on HRV-guided training but have followed training interventions based on a traditional and nonindividualized methodology.

In contrast, evidence exists supporting the use of HRV-guided training for improved performance in endurance athletes. With this type of training monitoring, some studies have found significant VO_2max_ improvements in athletes who have developed individualized endurance training programs based on daily HRV values. These studies alternated moderate-intensity sessions with high-intensity sessions [4,10] or even rest sessions, vigorous-intensity training, and moderate-intensity exercise [5]. However, Javaloyes et al. [18], in a program with similar characteristics developed with professional cyclists, found no significant improvements in VO_2max_. Likewise, significant improvements have been found among athletes following HRV-guided training in other variables; for example, for lactate in maximal test [10], speed in maximal test [4], time in maximal test [4,10], or muscle strength [24]. At the level of perceived recovery, significant improvements have also been found in variables such as general stress, emotional stress, lack of energy, and even overall mood disturbance [25]. HRV-guided training may, therefore, function as an alternative method for improving performance in resistance athletes.

### 1.4. Why Is This Review Important?

In the search to improve athletic performance, different training methods have been tried and studied, such as intensified training [2] or submaximal tests [26]. However, it has also been recognized that the same training program followed by a group of athletes can provoke a wide range of reactions in terms of performance and physiological adaptations [3]. Overuse injuries occur due to repetitive submaximal loading of the musculoskeletal system when there is inadequate rest to allow for structural adaptation to take place [27]. In recent years, HRV-guided training has shown itself to be a promising method for improving different performance variables (e.g., VO_2max_) compared to predefined training (traditional training) through the monitoring and individualization of endurance athletes’ training [4,28]. HRV-guided training has been investigated in randomized trials on samples from different endurance sports, such as skiers [28], runners [4,25], and cyclists [18]), as well as athletes of different ages and levels: elite [18,28] and recreational endurance athletes [5,24,25]. Therefore, it is important to carry out a systematic review and meta-analysis of the different experimental studies conducted so far on endurance athletes in order to assess whether HRV-guided training is an effective method for performance improvement.

## 2. Objectives

As mentioned above, this review aimed to analyze the effect of HRV-guided training on VO_2max_ in endurance athletes.

We asked the following research questions regarding HRV-guided training in endurance athletes:

**Research Question 1**: Does HRV-based training have an effect on VO_2max_?
**Research Question 2**: Is the effect of this type of training superior to that of traditional training?
**Research Question 3**: Is the level of the athletes decisive in obtaining an effect on the VO_2max_?
**Research Question 4**: Does the effect of HRV-guided training determine VO_2max_ scores according to the gender of the athlete?

## 3. Methods

The methods detailed below are reported in accordance with the Campbell Collaboration policies and guidelines for systematic reviews [29].

### 3.1. Criteria for Considering Studies for This Review (Eligibility Criteria)

#### 3.1.1. Types of Studies

We included randomized controlled trials (RCTs) and the first period of cross-over RCTs and experimental studies using a random method for the treatment assignment in order to reduce the risk of allocation bias. We restricted study eligibility by language. We did not restrict study eligibility by publication status.

#### 3.1.2. Types of Participants

We included studies with trained athletes enrolled in any form of regular endurance training (e.g., runners, triathletes, skiers, and cyclists). We included studies that recruited both men and women, or men and women separately.

#### 3.1.3. Types of Interventions

We included studies on endurance training controlled by heart rate variability to improve the athletes’ performance. We considered designs comprising any dose, frequency, and duration. We also considered studies with the following types of comparisons:

- Endurance training controlled by HRV versus no specific training intervention (e.g., habitual physical activity).
- Endurance training controlled by HRV versus another training intervention (e.g., traditional endurance training or another type of traditional training).
- Endurance training controlled by HRV versus another training intervention (i) versus a further training intervention (ii).
- Endurance training controlled by HRV (i) versus endurance training controlled by HRV (ii) versus another training intervention versus no specific training intervention.

#### 3.1.4. Types of Outcome Measures

- Primary
  ∘ Maximal oxygen consumption (VO_2max_)

### 3.2. Search Methods to Identify the Studies

#### 3.2.1. Electronic Searches

The register contains studies identified from the Cochrane Central Register of Controlled Trials (CENTRAL), MEDLINE, EMBASE, CINAHL Complete, the Web of Science Core Collection, Global Health, Current Contents Connect, and the SciELO citation index.

The search is up to date as of 15 June 2020. The language was restricted, considering only English or Spanish. The terms used to search the databases were: (*amateur* OR *elite* OR train*) AND (*HRV-guided* OR *“heart-rate variability guided”*).

#### 3.2.2. Searching Other Resources

We checked the reference lists of all the included studies and systematic reviews for additional references. We contacted experts in the field and the authors of the included studies to identify additional unpublished studies. We also checked the results of completed trials registered on the US National Institutes of Health Ongoing Trials Register, ClinicalTrials.gov, the World Health Organization International Clinical Trials Registry Platform (WHO ICTRP), and proceedings of conferences for relevant research.

### 3.3. Data Collection and Analysis

We conducted the following data collection and analysis in accordance with the recommendations in the Cochrane Handbook for Systematic Reviews of Interventions [30].

#### 3.3.1. Selection of Studies

Two review authors independently screened the titles and abstracts of all the retrieved references in Microsoft Excel 2018 (Microsoft, New York, NY, USA) for Windows. The full-text study reports were retrieved for all the citations that at least one review author considered potentially relevant. Two review authors independently screened the full-text articles and identified studies for inclusion; they also identified and recorded the reasons for excluding studies in the excluded studies characteristics. Any disagreements were resolved through discussion. The selection process is detailed in a PRISMA flow diagram [31].

#### 3.3.2. Data Extraction and Management

We used a standardized piloted data collection form in Microsoft Excel 2018 for Windows and extracted the following study characteristics and outcome data: (i) Methods: study design; (ii) Participants: randomized number, study participants’ mean age or age range, study location and setting, recruitment methods, inclusion and exclusion criteria, and type of endurance sport; (iii) Interventions: a description of the training intervention characteristics, the dose and duration of the training intervention, a description of the comparison intervention characteristics, the length of follow-up, the number of withdrawals, and the reasons for withdrawal; (iv) Outcomes: a description of the primary and secondary outcomes in the review that were reported in the trial and a listing of other outcomes collected in the trial; (v) Notes: the trial funding and notable conflicts of interest of the trial authors; (vi) a ‘risk of bias’ assessment. Two review authors independently extracted the outcome data from the included studies into Microsoft Excel 2018 spreadsheets and compared the data to identify any discrepancies in the data entries. Any disagreements were resolved by consensus. In the Characteristics of Included Studies section, we noted down if a trial did not report outcome data in a usable way. We then transferred all the outcome data into the Comprehensive Meta-Analysis software version 2.2.064 (Biostat, Englewood, NJ, USA) [32].

#### 3.3.3. Risk-of-Bias Assessment in the Included Studies

Two review authors (M.C.P., A.G.G.) independently assessed the risk of bias for each included trial using the Cochrane risk-of-bias tool [30]. Any disagreements were resolved by discussion. The risk of biases were assessed for the following domains: random sequence generation (selection bias), allocation concealment (selection bias), blinding of participants and personnel (performance bias), blinding of outcome assessment for each outcome (detection bias), incomplete outcome data (attrition bias), selective outcome reporting (reporting bias), and other biases (such as the validity of outcome measure and baseline comparability). Each potential source of bias was assessed as either high, low, or unclear, and a quotation from the study report was provided together with a justification for the judgment in the ‘risk of bias’ tables. The judgments across the different studies were summarized for each of the domains listed.

#### 3.3.4. Treatment Effect Measures

The outcome data for each study were uploaded into the data tables of the Comprehensive Meta-Analysis software to calculate the treatment effects. We used the mean difference (MD) for continuous outcomes reported on the same scal, and the standardized mean difference (SMD) for continuous outcomes measured on different scales in different trials (SMD = M_HRV guided training_ − M_control group_/Standard deviation) [33]. Uncertainty was expressed with 95% confidence intervals (CIs) for all the effect estimates.

#### 3.3.5. Assessment of Heterogeneity and Reporting Bias

Heterogeneity was assessed qualitatively between studies in three ways: a visual examination of the forest plots, the Chi^2^ test (*p* ≤ 0.10) for heterogeneity, and the I^2^ statistic. The implications of the observed I^2^ statistic value were considered as follows: 0% to 40%—might not be important; 30% to 60%—may represent moderate heterogeneity; 50% to 90%—may represent substantial heterogeneity; 75% to 100%—considerable heterogeneity [30]. Publication bias was assessed by examining the asymmetry of a funnel plot using Egger’s test. If studies were distributed symmetrically around the mean effect size (ES), there was an absence of publication bias [33]. Subgroup analysis was carried out using the outcome for athlete level (elite vs. amateur) and sex (men, women, and both sexes combined). Metaregression was used to assess the relationship between the studies and the variable sample size.

#### 3.3.6. Sensitivity Analysis

A sensitivity analysis was carried out to check whether the results varied according to the endpoint data.

## 4. Results

### 4.1. Description of the Studies

#### 4.1.1. Search Results

**Figure 1:**
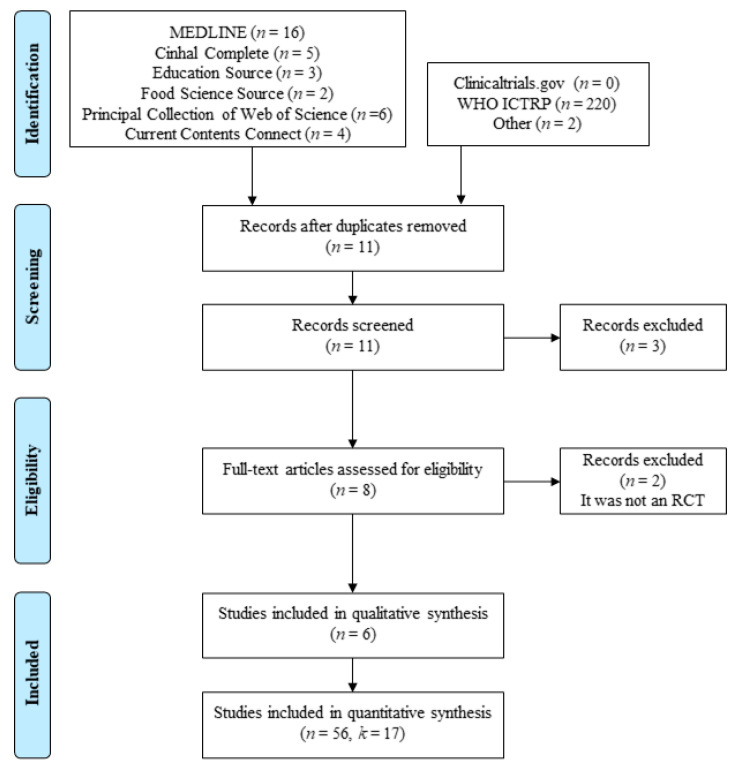
Study flow diagram following the Preferred Reporting Items for Systematic Reviews and Meta-Analyses Guidelines [31], where n is the number of papers and k is the number of individual studies.

The search produced a total of 36 studies, with 222 additional records identified through other sources. The removal of duplicates resulted in eleven studies, which were screened by the two authors based on the title and abstract. Three studies were excluded. Eight full-text articles were assessed for eligibility. Two more studies were excluded, and six studies were included either in the qualitative analysis or in the quantitative metasynthesis. The PRISMA flow chart illustrates the search and selection process (Figure 1).

#### 4.1.2. Included Studies

Six studies carrying out HRV-guided training with elite or amateur athletes were included in this review [4,5,10,18,19,28], which were identified by the first author and publication date: Javaloyes_2019, Kiviniemi_2007, Kiviniemi_2010, Nuuttila_2017, Schmitt_2018, and Vesterinen_2016.

- Study location

Schmitt_2018 conducted their study at the French National Ski-Nordic Center, while the locations for the other five studies were not specified.

- Study design

Every study included in this review was a randomized controlled trial.

- Participants

A total of 195 participants (134 men and 61 women) were included in these studies. Kiviniemi_2007, Javaloyes_2019, and Nuuttila_2017 considered only male samples of 30, 17, and 32 participants, respectively. In the rest of the studies, the samples were composed of men and women: Kiviniemi_2010 included 24 men and 36 women, Schmitt_2018 incorporated 19 men and 5 women, and Vesterinen_2016 assessed 20 men and 20 women. In the studies by Javaloyes_2019 and Schmitt_2018, the samples were composed of professional athletes (cyclists and Nordic skiers, respectively) while in the other four studies, the samples were of a nonprofessional level.

- Interventions

According to the types of comparisons contemplated in the present systematic review ((a) endurance training controlled by HRV versus no specific training intervention; (b) endurance training controlled by HRV versus other training intervention; (c) endurance training controlled by HRV (i) versus another training intervention (ii) versus another training intervention; (d) endurance training controlled by HRV (i) versus endurance training controlled by HRV (ii) versus other training intervention versus no specific training intervention. Kiviniemi_2007, Javaloyes_2019, Nuuttila_2017, and Vesterinen_2016 were classified in Comparison B, Schmitt_2018 in Comparison C, and Kiviniemi_2010 in Comparison D.

The interventions in the included studies focused on running (Kiviniemi_2007, Kiviniemi_2010, Nuuttila_2017, and Vesterinen_2016), skiing (Schmitt_2018), and cycling (Javaloyes_2019). They were from 6 to 15 weeks long. In most of the studies, three (Nuuttila_2017) or four (Javaloyes_2019, Schmitt_2018, and Vesterinen_2016) low-intensity preparation weeks were followed either by the experimental or control groups (standard training) before the intervention. An eight-week intervention was carried out in Javaloyes_2019, Kiviniemi_2010, Nuuttila_2017, and Vesterinen_2016, whereas Kiviniemi_2007 considered four weeks of training and Schmitt_2018 15 days. The assessment weeks were treated separately from the intervention period in Javaloyes_2019, Kiviniemi_2007, and Schmitt_2018.

In every study, the experimental groups trained at moderate or high intensities according to their daily HRV scores. The control groups (standard training) followed a predefined training design at high, moderate, and low intensities (Javaloyes_2019), high and moderate intensities (Kiviniemi_2010 and Nuuttila_2017), high and low intensities (Kiviniemi, 2007) or moderate and low intensities (Vesterinen_2016). The control group (standard training) design was not explained in Schmitt_2018.

- Outcomes

The primary outcome analyzed in the included studies was VO_2max_. The secondary outcomes were: ventilatory thresholds (Javaloyes_2019, Kiviniemi_2007) and power in the cycling test (Javaloyes_2019); rMSSD or RR interval (Javaloyes_2019, Kiviniemi_2007, and Schmitt_2018); basal heart rate (Nuuttila_2017, Kiviniemi_2010, and Schmitt_2018); maximal heart rate in the ergometer test (Nuuttila_2017); speed in the treadmill test (Kiviniemi_2007, Nuuttila_2017, and Vesterinen_2016); maximal speed in the 10 m test (Nuuttila_2017); time and lactate in the 3000 m test (Nuuttila_2017); maximal load in the ergometer test (Kiviniemi_2007 and Kiviniemi_2010); and oxygen saturation and VO_2_ at the second ventilatory threshold (Schmitt_2018).

Further details about participants, interventions, comparators, and outcomes are provided in Table 1.

#### 4.1.3. Excluded Studies

As indicated in Figure 1, five studies were excluded from the qualitative analysis. Three studies were excluded because the VO_2max_ was not considered as an outcome [24,25,34], and two studies were excluded because they were not RCTs [35,36].

### 4.2. Risk of Bias in the Included Studies

**Figure 2:**
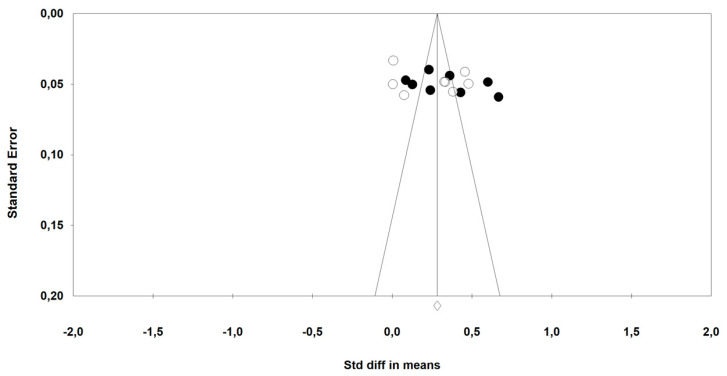
Funnel plot of standard error by standard differences in means (17 comparison; black circle, HRV-guided training; white circle, traditional training).

The risk of bias in the included studies is summarized in Table 2. This assessment was made following the Cochrane Collaboration guidelines [30]. In addition, publication bias was assessed using a funnel plot (Figure 2). The Egger test provided statistical evidence of funnel plot symmetry, suggesting the absence of a significant publication bias (*p* = 0.101).

- Selection bias

In Javaloyes_2019, Kiviniemi_2007, and Kiviniemi_2010, neither the random component in the sequence generation nor the allocation concealment were described; therefore, the risk-of-bias selection was considered unclear. In Nuuttila_2017, Schmitt_2018, and Vesterinen_2016, the risk of bias was considered high because the randomization sequence was, in the first stage, based on the results of certain physical condition tests, sport discipline, age, or gender. Furthermore, in the second stage, the random component or the allocation concealment was not described.

- Performance and detection bias

The detection bias was considered unclear in all of the included studies because they did not address this outcome. The performance bias was also unclear in every study but Javaloyes_2019, which was considered high because only the participants were blinded, thus the blinding was incomplete.

- Attrition bias

In Javaloyes_2019 and Schmitt_2018, the attrition bias was considered low because there were no missing outcome data. On the other hand, Kiviniemi_2010, Nuuttila_2017, and Vesterinen_2016 presented high rates of follow-up loss for different reasons. These might be relevant in the ES observed. Moreover, no statistical procedure, such as intention-to-treat, was used to minimize this risk of bias. Therefore, they were considered as having a high risk of attrition bias. Finally, in Kiviniemi_2007, the attrition bias was unclear because this outcome was not addressed in the study.

- Reporting bias

The study protocols for the included studies were not available. Accordingly, Javaloyes_2019, Kiviniemi_2007, Kiviniemi_2010, and Nuuttila_2017 were considered as having an unclear reporting bias. For their part, Schmitt_2018 and Vesterinen_2016 did not report every outcome and were thus considered as having a high risk of reporting bias.

- Other biases

The included studies appear to be free from other sources of bias.

### 4.3. Synthesis of Results

The Kiviniemi_2010 and Schmitt_2017 studies were segmented for quantitative analysis according to their intervention groups. The comparisons were: Kiviniemi_2007 a, HRV (male subgroup, HRV-guided training) vs. standard training (ST); Kiviniemi_2010 a, HRV-1 (male subgroup, HRV-guided training) vs. standard training (ST); Kiviniemi_2010 c, HRV-I (female subgroup, HRV-guided training) vs. standard training (ST); Kiviniemi_2010 g, HRV-II (female subgroup, HRV-guided training tailored for women) vs. HRV-I (female subgroup, HRV-guided training); Kiviniemi_2010 f, HRV-II (female subgroup, HRV-guided training tailored for women) vs. standard training (ST); Schmitt_2017 a HRV (HRV-guided training) vs. N (traditional training and normoxia sleeping); Schmitt_2017 b HRV (HRV-guided training) vs. H (traditional training and hypoxia sleeping). Therefore, the total number of individual studies analyzed were 17 (k = 7 for the experimental group; k = 10 for the control group).

- Primary outcome measures

**Figure 3:**
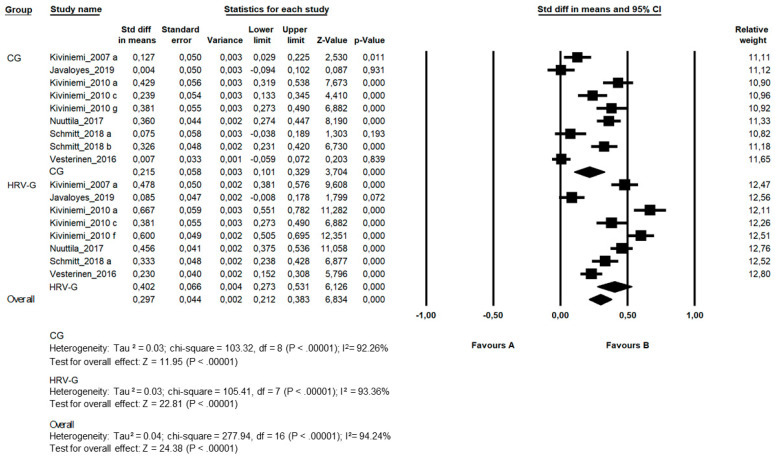
Standard differences in means (SDM) between post- and premeasures for VO_2max_ in included studies, segmented by the control group (CG) and heart-rate-variability-guided training group (HRV-G). Squares represent the SDM for each trial; the diamond represents the pooled SDM across trials; weight determines how much each individual study contributes to the pooled estimate; 95%CI, confidence interval.

There were five studies (Kiviniemi_2007, Kiviniemi_2010, Nuuttila_2017, Schmitt_2017 and Vesterinen_2016) with significant intragroup VO_2max_ improvements in the HRV-guided training group (*n* = 95), while no significant changes were found in Javaloyes_2019 (*n* = 9). On the other hand, in three studies (Kiviniemi_2010, Nuuttila_2017, and Vesterinen_2016), there were also significant intragroup VO_2max_ improvements in the control group (*n* = 47). The overall risk of bias was considered high in every study but for Javaloyes_2019, which was considered unclear. A random-effects meta-analysis of the six studies revealed a statistically significant (*p* < 0.0001) treatment effect for VO_2max_ in the HRV-guided training intervention (ES = 0.402; 95%CI = 0.273, 0.531). Moreover, the other training intervention was also statistically beneficial (*p* < 0.0001) for VO_2max_ improvements in the control group (ES = 0.215; 95% CI = 0.101, 0.329). However, the ES for the VO_2max_ was significantly higher (*p* < 0.0001) in the HRV-guided training group. The heterogeneity observed in the meta-analysis was significant and high in the overall analysis (*p* < 0.0001; I^2^ = 94.24%) and for the experimental (*p* < 0.0001; I^2^ = 9.36%) and the control group (*p* < 0.0001; I^2^ = 92.26%) (Figure 3).

- Moderator analyses

**Figure 4:**
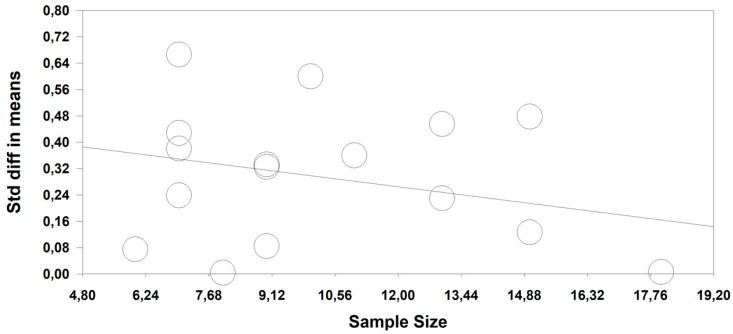
Metaregression of the number of participants (sample size) on standard differences in means (Std diff in means).

Owing to the high heterogeneity observed in the meta-analysis, the potential moderating effect of the following was considered to be of interest: (a) the athletes’ level (elite vs. amateur) and (b) the sex of the participants (‘men vs. women’ vs. ‘men and women’). We had originally planned to take into account the intervention duration; however, it was not finally included as a subgroup owing to there being only one study that considered an intervention period of 15 days (Schmitt_2017) while the others conducted an eight-week intervention. The sample size was used for the metaregression. Following the moderating variables (Table 3), the athletes’ level (elite vs. amateur) brought about statistically significant improvements (*p* < 0.0001) in both subgroups, while there were statistically significant differences between the subgroups (*p* < 0.0001) in favor of the nonprofessional subgroup (elite, ES = 0.17; amateur, ES = 0.36). According to the sex subgroups (‘men vs. women’ vs. ‘men and women’), there were statistically significant improvements (*p* < 0.0001) in the three subgroups and statistically significant differences (*p* < 0.0001) between the three subgroups in favor of the women (men, ES = 0.33; women, ES = 0.40; men and women, ES = 0.19). The metaregression findings (Figure 4) revealed that the sample size of the studies was directly related to the ES magnitude (regression coefficient = −0.016; standard error = 0.003; lower limit = −0.023; upper Limit = −0.011; Z-value = −5.42; *p* ≤ 0.0001).

## 5. Discussion

### 5.1. Summary of Main Results

Six RCT studies evaluating the effects of an HRV-guided training intervention on endurance athletes were included in this review. The results of the meta-analyses provide some evidence that either HRV-guided training or traditional training may improve their performance in terms of VO_2max_ (HRV-G: ES = 0.402, *p* < 0.0001; CG: ES = 0.215, *p* < 0.0001). However, more favorable outcomes (*p* < 0.0001) for the experimental groups compared to the control groups were recorded across the studies. Moderators indicated larger effect sizes for interventions involving amateur endurance athletes (ES = 0.36, *p* < 0.0001) and women (ES = 0.40, *p* < 0.0001). On the other hand, the sample size of the studies was directly related to the ES magnitude (*p* < 0.0001).

### 5.2. Overall Completeness and Applicability of the Evidence

The total sample size of the studies meeting our original inclusion criteria was sufficiently large to warrant restricting the results to a meta-analysis of the RCTs. Data on the primary outcome (VO_2max_) were measured directly using a gas exchange analysis system and a maximal test in each study. This is the most accurate way to obtain cardiorespiratory data. However, some studies implemented this test using a treadmill (Kiviniemi_2007, Nuuttila_2017, Schmitt_2017, and Vesterinen_2016) and others using a cycle ergometer (Javaloyes_2019 and Kiviniemi_2010). In the first case, training was based on running (Kiviniemi_2007, Nuuttila_2017, and Vesterinen_2016) and skiing (Schmitt_2017), which implies similar technical execution in the test. In the second case, the Javaloyes_2019 study was carried out on cyclists, whereas the Kiviniemi_2010 study sample was composed of runners. Statistical improvements regarding VO_2max_ were found in the Kiviniemi_2007 and Kiviniemi_2010 studies. However, the specificity of the test may be a source of variability and potential imprecision in the second study results. Following the training specificity principle [37], the body’s physiological and metabolic responses and training adaptations are specific to the type of exercise and the muscle groups involved. Thus, the evaluation method should be as similar as possible to the training in order to obtain the most reliable results. This needs to be taken into account when interpreting the results.

Despite the intervention durations being quite homogeneous in the included studies (eight weeks for each study apart from Kiviniemi_2007 and Schmitt_2017), the total duration of the training process, preparation weeks included, endurance sport modality, and training intensities used for the control group (standard training) were different. There was also a marked heterogeneity in the sample of the included studies: elite (Javaloyes_2019 and Schmitt_2017) and amateur (Kiviniemi_2007, Kiviniemi_2010, Nuuttila_2017, and Vesterinen_2016) participants, or samples comprising only men (Javaloyes_2019, Kiviniemi_2007, Kiviniemi_2010, and Nuuttila_2017), women (Kiviniemi_2010), or men and women (Schmitt_2017 and Vesterinen_2016). A standardized training protocol should be recommended to ensure the optimal benefits regarding VO_2max_.

### 5.3. Quality of the Evidence

The quality of the evidence from the included studies can be considered unclear. Despite each study being a randomized controlled trial, the sequence generation or the allocation concealment was considered skewed in half of them. The performance bias was high only in Javaloyes_20019, while the detection bias was unclear in all the studies because incomplete blinding was considered. Attrition was high in Kiviniemi_2010, Nuuttila_2017, and Vesterinen_2016 because of the high follow-up rates. In addition, the reporting bias was generally unclear due to the lack of a registered protocol.

### 5.4. Potential Biases in the Review Process

Although the systematic nature of the review process followed here decreases the potential for bias, the risk of bias in the review process remains. The greatest risk of bias present in this review was the study selection; specifically, the decision to limit the inclusion criteria to individual endurance sports, thus reducing the number of studies included and causing a potential limitation in the results.

- Agreements and disagreements with other studies or reviews

Based on the results from this systematic review with meta-analysis, and in response to Research Question 1, it is not surprising that the meta-analyzed results regarding improvements in athletes’ VO_2max_ were associated with both training methodologies. According to Bartlett, O’Connor, Pitchford, Torres-Ronda, and Robertson [2] and Heyward [37], adequate prescribed training should maximize athletic performance when the specificity, overload, progression, initial level, individualization, diminishing return, and reversibility principles are followed. However, it was also found that the individual training adaptation according to the endurance athletes’ daily HRV scores produced better VO_2max_ results than the standardized prescribed training, which answers Research Question 2. As pointed out by Vesterinen et al. [4,9], not every athlete improves their VO_2max_ after standardized group training. Similarly, Gallo, Cormack, Gabbett, Williams, and Lorenzen [38] reported that, in footballers, the internal load (perceived effort) of each athlete was different for a given external load; this definitely affects their individual performance during training and will be reflected in their individual performance improvements. Thus, daily individual HRV monitoring and training guidance balancing the sympathetic and parasympathetic autonomic nervous system leads to greater athletic performance in endurance athletes compared to standardized prescribed training. This is relevant if training optimization is the objective, supporting the idea that training should be prescribed appropriately to avoid overtraining and/or injury [38]. In the same vein, it is also interesting to point out that, according to studies such as Hulin, Gabbett, Lawson, Caputi, and Sampson [39] and Williams et al. [16], training individualization is also related to minimizing overuse and reducing the injury risk, which may be a correlative benefit in the pursuit of endurance athlete training optimization.

On the other hand, the meta-analyzed results show that VO_2max_ improvements were greater when the sample comprised amateur endurance athletes. This answers Research Question 3. According to the initial training level principle [37], individuals with a low initial level of physical fitness should achieve more significant relative increases than those of average or high levels. This is in accordance with the results of Sanchez-Sanchez et al. [40], where greater performance improvements were obtained in lower-level football players compared to the higher-level players, concluding that the lower the athlete’s initial fitness level, the higher the available window of adaptability. Conversely, in the systematic review with meta-analysis by Hammami, Gabbett, Slimani, and Bouhlel [41], the athlete’s level was not a determinant variable in terms of VO_2max_ enhancement since it improved if they were elite or amateur players. It should be noted that this review was conducted on football players and that randomized and nonrandomized controlled trials were included.

According to our meta-analyzed results, and in response to Research Question 4, there were higher effect sizes regarding VO _2max_ improvements when the sample was not mixed, especially in the case of women. There is controversy concerning the influence of sex on sport performance. Recent studies conducted on endurance athletes concluded that either sex was not a predictor variable of performance [42] or that performance between men and women was different in swimming, cycling, and running [43]. In the case of the present systematic review with meta-analyses, we consider that the initial level of the sample influenced the result, given that, in the Kiviniemi_2010 study, when female samples were analyzed, the participants were amateur level athletes. Thus, a higher relative performance increment is predictable based on the athletes’ level.

## 6. Conclusions

### 6.1. Practical Implications

Training optimization to enhance performance in endurance athletes is a goal that is undergoing a constant process of improvement. Finding a procedure to objectively individualize the training would be ideal for achieving this goal. The meta-analyses results considered in this review suggest that HRV is a good indicator of physiological responses to training in endurance athletes. Consequently, using daily HRV scores for training individualization and prescription is an effective method for optimizing performance in endurance athletes. This is reflected in the improved VO_2max_ results when the training is guided by HRV, considering VO_2max_ as one of the main performance indicators. In addition, it should be taken into account that a lower initial athlete fitness level will be relevant in achieving greater VO_2max_ improvement. Although gender may be a variable that influences the performance gains, in our opinion, this result is primarily conditioned by the level of the athletes included in the analyzed studies. Therefore, we do not consider it to be a variable that clearly affects VO_2max_ improvements.

### 6.2. Research Implications

The results from this review suggest that, while there is evidence that HRV-guided training is effective at improving VO_2max_ in endurance athletes, there is still work to be done in terms of identifying the characteristics of the interventions that contribute to this effect and the characteristics of participants who are more likely to respond to such interventions. The most important point is that more research is required since only five studies were included in this review. Moreover, only two of the studies used samples composed of elite endurance athletes, which gave different results regarding VO_2max_ improvement. Consequently, the research should be extended to the professional field in order to clarify the effect of guiding training on VO_2max_. This would also help to clarify whether the endurance sport modality is determinative of the VO_2max_ enhancement when following this training methodology.

Using daily HRV scores to control the training load and intensity over eight weeks is enough to improve VO_2max_ in endurance athletes. Nonetheless, the training protocol should be further standardized in terms of adjusting the number of preparation weeks or considering the measurement weeks within or around the training period, factors that determine the training duration. Moreover, the standardized training protocol used in the control groups varied between the studies, which considered low, moderate, or high training intensities, as well as different numbers of sessions per week and session durations. This might very well have influenced the VO_2max_ results. Therefore, it is necessary to reach a consensus regarding a standardized training protocol to use in future studies. In this line, it has been recently published a protocol [44] that could clarify the studies design. Similarly, although each study in this review used the most accurate method available to obtain the cardiorespiratory data, in the future, we should consider using a measuring instrument that allows us to implement the most specific sport technique in order to minimize result variability and imprecision.

Regarding the quality of the studies, authors should consider: improving the sequence generation or allocation concealment, the blinding of the participants, personnel, and outcome assessors, the rates of follow-up loss, using statistical procedures such as intention-to-treat to minimize attrition bias, and registering their protocols before starting the randomized controlled trial.

Lastly, to reinforce knowledge regarding performance optimization in endurance athletes, a good way to supplement the effect of HRV-guided training might be to register the risk of injuries associated with overuse using tools such as the Oslo Sports Trauma Research Center Overuse Injury Questionnaire, since this considers additional aspects affecting the execution of athletes’ training.

## Figures and Tables

**Table 1:** Overview of the studies included in the review.

Author, Year	Method	Participants	Intervention	Outcomes	Results	Risk of Bias		
						Bias	Author’s Judgment	Support for the Judgment
Javaloyes_2019	Randomized controlled trial	Trained male cyclist, mean age of 38.42 years. *N* = 17: EG = 9 + CG = 8 Location: not specified. Recruited from local clubs Inclusion criteria: at least 2 years of experience in cycling. Exclusion criteria: not specified.	15 weeks (4 weeks of baseline period to capture baseline HRV + 8 weeks of training + 3 weeks of assessments); 4–7 sessions/week; time depended on the training intensity. EG: HRV-G-based training before each session; training MICT and HIIT according to HRV. CG: 4 high-intensity training sessions + 4 high-intensity interval training sessions + 6 moderate-intensity training sessions + 2–5 low-intensity training sessions/week. No follow-up periods. No withdrawals	Primary: VO_2max_ (maximal bicycle ergometer test, direct measurement). Secondary: ventilatory thresholds in the graded test, peak power output in the graded test, rMSSD with a heart rate monitor + kubios (LnrMSSD) in a supine position for 90 s, mean power output during a 40 min all-out cycling test.	VO_2max_: no significant differences between intragroups and intergroups. Moderate training load: significant intergroup differences (EG = 24%; CG = 27%). VT2: significant improvements in EG (36.11 ± 3.73W). Peak power output: significant improvements in EG (17.45 ± 3.91W). LnrMSSD: significant differences between intergroups for the percentage of change (EG = 0.85 ± 3.21%, CG = –2.02 ± 5.21%). Mean power 40M: significant improvements in EG (17.67 ± 3.03W)	Selection	Unclear	Insufficient information about the sequence generation process and allocation to permit judgment of ‘low risk’ or ‘high risk’.
						Performance	High	Incomplete blinding, and the outcome is likely to be influenced by lack of blinding.
						Detection	Unclear	The study did not address this outcome.
						Attrition	Low	No missing outcome data.
						Reporting	Unclear	Insufficient information to permit judgment of ‘low risk’ or ‘high risk’.
						Other	Low	The study appears to be free of other sources of bias.
Kiviniemi_2007	Randomized controlled trial	30 healthy recreational male runners *N* = 30: TRA: predefined training group (*n* = 10) + HRV: HRV-guided training (*n* = 10) + CG: Control group (*n* = 10). Location: Not specified Recruitment: The candidates were interviewed with a standardized scheme to ascertain their medical history and levels of physical activity. Inclusion criteria: healthy men. Exclusion criteria: subjects who had done regular physical exercise training less than twice a week for the past 3 months, competing athletes, and subjects with diabetes mellitus, asthma, or cardiovascular disorders were excluded.	6 weeks: 1-week baseline resting + pretest Intervention: 4-week training period (6 days per week) consisting of running sessions at either a low- or high-intensity level according to recommendations by the American College of Sports Medicine: low-intensity: 40 min of jogging at 65% of maximal HR; high-intensity exercise included 5 min warm-up and cool-down periods at 65% of the maximal HR before and after 30 min of running at 85% of maximal HR. The last week for the post-test. HRV: exercised at low- or high-intensity or rested based on their daily HRV measurements at home. If HRV increased or did not change, vigorous-intensity training on that day. If HRV decreased, moderate-intensity exercise or rest. TRA: weekly training started with low-intensity exercise followed by two sessions of high-intensity exercise on successive days. This 3-day period was repeated before a day of rest. CG: no intervention No follow-up period. 4 withdrawals: TRA (2); HRV (1); CG (1).	Primary: VO2peak (maximal treadmill ergometer test: direct measurement). Secondary: high-frequency power of RR interval with software while standing for 5 min, maximal load in the ergometer test, maximal running velocity in the ergometer test; ventilatory threshold (VT) from the relation of running velocity and selected ventilatory parameters.	VO2peak: significant intragroup improvements in the HRV group (pretest = 56 ± 4; post-test = 60 ± 5 mL/kg/min). High-frequency power of RR interval: significant intragroup improvements in TRA (pretests = 4.7 ± 0.4; post-test = 5.5 ± 0.8 ln ms^2^), and HRV (pretests = 4.8 ± 0.6; post-test = 5.2 ± 0.8 ln ms^2^). Maximal load: significant intragroup improvements in TRA (pretest = 15.1 ± 1.3 km/h; post-test = 15.7 ± 1.2 km/h); significant intergroup differences between CG (post-test = 14.9 ± 1.5 km/h) and TRA (post-test = 15.7 ± 1.2 km/h), TRA and HRV (post-test = 16.4 ± 1.0 km/h), and between CG and HRV. VT: significant intragroup improvements in HRV (pretest = 12.2 ± 0.6 km/h; post-test = 16.4 ± 1.0 km/h)	Selection Performance Detection Attrition Reporting Other	Unclear Unclear Unclear Unclear Unclear Low	Insufficient information about the sequence generation process and allocation to permit judgment of ‘low risk’ or ‘high risk’. Insufficient information to permit judgment of ‘low risk’ or ‘high risk’. Insufficient information to permit judgment of ‘low risk’ or ‘high risk’. The study did not address this outcome. Insufficient information to permit judgment of ‘low risk’ or ‘high risk’ The study appears to be free of other sources of bias.
Kiviniemi_2010	Randomized controlled trial	Healthy men and women. Mean age of 34.57 years. *N* = 60. Men, *n* = 24; women, *n* = 36). ST: standard training (8 men + 8 women) + HRV-I: HRV-guided training for men and women (EG: 8 men + 8 women) + HRV-II: HRV-guided training tailored for women (12) + CG (8 men + 8 women). Location: Not specified Recruitment: advertisement local newspaper Inclusion criteria: healthy men and women Exclusion criteria: smoker, BMI ≥ 30 kg/m^2^; regular physical exercise training more than twice a week for the last 3 months, competing athletes, mellitus, asthma, or cardiovascular disorders.	8 weeks of aerobic exercise sessions (40 min), vigorous-intensity level: HR between 85% of the HRpeak-5 bpm lower limit; moderate-intensity exercise was 70% of the HRpeak-5 bpm lower limit. HRV-I: if HRV increased or did not change, vigorous-intensity training on that day. If HRV decreased, moderate-intensity exercise or rest. HRV-II: vigorous-intensity exercise only when HRV had increased. ST group: two moderate-intensity and three vigorous-intensity exercises weekly. CG: no intervention No follow-up period. 7 withdrawals: ST (1 man + 1 woman) + HRV-I (7 men + 7 women); CG (7 men + 8 women) + HRV-II (10); 4 because of illness or injury and 3 because of insufficient compliance.	Primary: VO_2max_ (maximal bicycle ergometer test: direct measurement). Secondary: HR, RR interval with a heart rate monitor (SD1) while standing for 3 min, maximal load in the ergometer test.	VO_2max_: significant intragroup improvements in ST (men subgroup) (pretest = 50 ± 7; post-test = 53 ± 7 mL/kg/min), ST (women subgroup) (pretest = 35 ± 5; post-test = 37 ± 4 mL/kg/min), HRV-I (men subgroup) (pretest = 50 ± 6; post-test = 54 ± 6 mL/kg/min), HRV-I (women subgroup) (pretest = 36 ± 4; post-test = 39 ± 3 mL/kg/min), and in HRV-II (women subgroup) (pretest = 37 ± 5; post-test = 40 ± 5 mL/kg/min). HR: RR interval: significant intragroup improvements in HRV-I (men subgroup) (pretests = 13.7 ± 6.7; post-test = 16.9 ± 8.7 ms). Maximal load: significant intragroup improvements in ST (men subgroup) (pretest = 275 ± 28W; post-test = 293 ±35W), ST (women subgroup) (pretest = 179 ± 32W; post-test = 198 ± 35W), HRV-I (men subgroup) (pretest = 270 ± 29W; post-test = 300 ± 25W), HRV-I (women subgroup) (pretest = 174 ± 28W; post-test = 189 ± 25W), and in HRV-II (women subgroup) (pretest = 177 ± 26W; post-test = 194 ± 23W)	Selection	High	Allocation based on the results of a laboratory test or a series of tests.
						Performance	Unclear	Insufficient information to permit judgment of ‘low risk’ or ‘high risk’.
						Detection	Unclear	The study did not address this outcome.
						Attrition	High	High rates of loss to follow-up.
						Reporting	Unclear	Insufficient information to permit judgment of ‘low risk’ or ‘high risk’.
						Other	Low	The study appears to be free of other sources of bias.
Nuuttila_2017	Randomized controlled trial	Males, 19–37 years. *N* = 24. EG = 13 and CG = 11. Location: not specified. Recruitment: not specified. Inclusion criteria: recreationally endurance training. Exclusion criteria: not specified	11 weeks (3 weeks of control + 8 weeks of training). EG: 2–5 sessions/week; CG: 6 sessions/week; time depended on the training intensity. EG: 4 moderate-intensity endurance training sessions + 20 high-interval intensity training sessions. Training MICT and HIIT according to HRV. CG: 22 moderate-intensity endurance training sessions + 20 high-interval intensity training sessions + 4 high-intensity strength training sessions No follow-up periods. 9 withdrawals: illness (*n* = 1), injuries (*n* = 2), personal reasons (*n* = 3), lack of adherence (*n* = 3).	Primary: VO_2max_ (maximal treadmill test: direct measurement). Secondary: basal heart rate, maximal heart rate, lactate in the treadmill test, Vmax in the treadmill test, Vmax in the 10m test, time and lactate in the 3000 m test, rMSSD with a heart rate monitor + Firstbeat in a supine position for 3 min. Other: body weight, height in countermovement jump, strength in the concentric dynamic leg press, nocturnal heart rate variability, testosterone, and cortisol (blood samples), % of fat (InBody 720).	VO_2max_: significant intragroup changes (EG = 3.1 ± 0.8 mL/kg/min; CG = 2.2 ± 0.6 mL/kg/min). Basal HR: significant intragroup decrease (EG = 4.4 ± 0.6bpm; CG = 3.6 ± 0.1bpm). Vmax in the treadmill test: significant intragroup improvements (EG = 0.9 ± 0.1km/h; CG = 0.5 ± 0.1 km/h). Vmax in 10 m: decreased significantly in CG from pre to mead test (0.08 ± 0.04 m/s). Time in the 3000 m test: significant intragroup decrease (EG = 35 ± 2s; CG = 35 ± 6s). Lactate in the 3000 m test: significant intragroup improvements (EG = 12 ± 18.4%) from mead-to post-test; CG = 16–0 ± 23.5%) from pre-to post-test. rMSSD: significant improvements in EG (13 ± 3ms)	Selection	High	Allocation based on the results of a laboratory test or a series of tests.
						Performance	Unclear	Insufficient information to permit judgment of ‘low risk’ or ‘high risk’.
						Detection	Unclear	The study did not address this outcome.
						Attrition	High	High rates of loss to follow-up.
						Reporting	Unclear	Insufficient information to permit judgment of ‘low risk’ or ‘high risk’.
						Other	Low	The study appears to be free of other sources of bias.
Schmitt_2018	Randomized controlled trial	24 elite Nordic skiers (19 men, age 23.3 ± 3.6; 5 women, age 22.8 ± 4.1). *N* = 24; H-HRV, HRV-guided training normobaric hypoxic group (n = 9) + H, sleeping in normobaric hypoxia group (n = 9); N, normoxia group (*n* = 6). Location: French National Ski-Nordic Center. Recruitment: members of the cross-country ski and Nordic combined French. Inclusion criteria: elite Nordic skiers. Exclusion criteria: a history of altitude-related sickness and health risks that could compromise the subject’s safety during training and/or hypoxic exposure.	Prior to pretest: 3 low-intensity training weeks (base training) with progressive training volume + 1-week recovery; Intervention: pretest + 15 days training (training load was organized into four training zones depending on the intensity and quantified as in Mujika et al. (1996), adapted to Nordic skiing (the threshold for training adjustment was chosen as 30% of the mean of the previous day) + postest1 + 1 week + postest2. Similar training content for each group. H-HRV group: sleeping normobaric in hypoxia (simulated altitude of 2700 m) with HRV-guided training; daily hypoxic dose was similar between H-HRV and H; Night SpO2 was similar between H-HRV and H, but lower than in N. H: traditional training sleeping in hypoxia (simulated altitude of 2700 m). N: traditional training sleeping in normoxia. Follow-up (post-test21 after 3 weeks of end postest1)	Primary: VO_2max_ (maximal treadmill test: direct measurement). Secondary: basal HR, peripheral oxygen saturation (SpO2), RR interval with a heart rate monitor (HF and LF) 5 min in a supine position and 5 min in standing, VO2 at the second ventilatory threshold. Others: duration of hypoxic exposure, HR, blood parameters (erythrocyte concentration, hemoglobin, hematocrit, ferritin), questionnaire of overtraining.	VO_2max_: significant intragroup changes in H-HRV (3.8 ± 3.1%). Basal HR: significant intergroup differences (H-HRV = 55.38 ± 10.02 vs. H = 55.59 ± 4bpm; H-HRV = 55.38 ± 10.02 vs. N = 47.11 ± 6.21bpm). SpO2: significant intergroup differences (H-HRV = 90.4 ± 1.3 vs. N = 94.2 ± 0.8%). RR interval: no significant differences between intergroups (H-HRV = 9561.10 ± 9436.02 ms^2^; H = 12,199.41 ± 1293.14 ms^2^; N = 7441.2 ± 4954.16 ms^2^). VO2 second VT: significant intragroup changes for H-HRV (6.7 ± 6.1%).	Selection	High	Allocation based on the results of a laboratory test or a series of tests.
						Performance	Unclear	Insufficient information to permit judgment of ‘low risk’ or ‘high risk’.
						Detection	Unclear	The study did not address this outcome.
						Attrition	Low	No missing outcome data.
						Reporting	High	Not all of the study’s prespecified primary outcomes have been reported.
						Other	Low	The study appears to be free of other sources of bias.
Vesterinen_2016	Randomized controlled trial	Recreational endurance runners (men = 20; women = 20) *N* = 40: EXP = 20 + TRAD = 20 Location: not specified. Recruitment: advertisement and social media Inclusion criteria: 2 years’ regular endurance running training. Exclusion criteria: disease or regular medication for chronic or long-term diseases.	12 weeks (4 weeks of preparation + 8 weeks of training). The same volume as before the study for PREP and the same volume as for PREP for INT. EXP: training MICT and HIIT according to HRV. TRAD: 50% sessions at low-intensity and 50% sessions at moderate/high-intensity. Week periodization, 3:1. No follow-up periods. 9 withdrawals: sicknesses (*n* = 2), injuries (*n* = 2), lack of adherence (*n* = 5)	Primary: VO_2max_ (maximal treadmill test: direct measurement). Secondary: Speed in Lactate 1, speed in Lactate 2, mean speed in the 3000 m test, time in the 3000 m test, RR intervals (rMSSD) 4 min in a supine position.	VO_2max_: significant intragroup improvements (EXP = 3.7 ± 4.6%, TRAD = 5.0 ± 5.2%). Speed in L1 significant intragroups improvement in EXP (2.8 ± 3.7%). Speed in L2 significant intragroups improvement in EXP (2.6 ± 3.3%) and TRAD (1.9 ± 2.2%). Time in the 3000 m test: significant intragroup improvements in EXP (–14.3 ± 14.1 s)	Selection	High	Allocation based on the results of a laboratory test or a series of tests.
						Performance	Unclear	Insufficient information to permit judgment of ‘low risk’ or ‘high risk’.
						Detection	Unclear	The study did not address this outcome.
						Attrition	High	High rates of loss to follow-up.
						Reporting	High	Not all of the study’s prespecified primary outcomes have been reported.
						Other	Low	The study appears to be free of other sources of bias.

**Table 2:** Risk of bias in the included studies.

Study	Risk-of-Bias Domains						
	Selection	Performance	Detection	Attrition	Reporting	Other	Overall Risk of Bias
Javaloyes_2019	Unclear	High	Unclear	Low	Unclear	Low	Unclear
Kiviniemi_2007	Unclear	Unclear	Unclear	Unclear	Unclear	Low	Unclear
Kiviniemi_2010	Unclear	Unclear	Unclear	High	Unclear	Low	Unclear
Nuuttila_2017	High	Unclear	Unclear	High	Unclear	Low	Unclear
Schmitt_2018	High	Unclear	Unclear	Low	High	Low	Unclear
Vesterinen_2016	High	Unclear	Unclear	High	High	Low	Unclear

**Table 3:** Subgroup analyses for measuring their impact on VO_2max_.

Research Studies			Variable: VO_2max_			
Group	No Studies	References	SMD (95% CI)	I^2^	*p*	*p*-Difference
Athlete level						
Elite	3	Javaloyes_2019; Schmitt_2018 a; Schmitt_2018 b	0.17 (0.03; 0.30)	89.63	<0.001	<0.001
Amateur	5	Kiviniemi_2010 a; Kiviniemi_2007 a; Kiviniemi_2010 c; Kiviniemi_2010 g; Nuuttila_2017; Vesterinen_2016	0.36 (0.24; 0.48)	94.66	<0.001	
*Sex*						
Women	3	Kiviniemi_2010 c; Kiviniemi_2010 f; Kiviniemi_2010 g	0.40 (0.25; 0.56)	88.36	<0.001	<0.001
Men	4	Javaloyes_2019; Kiviniemi_2007 a; Kiviniemi_2010 a; Nuuttila_2017	0.33 (0.17; 0.48)	94.98	<0.001	
Men and women	3	Schmitt_2017 a; Schmitt_2017 b; Vesterinen_2016	0.19 (0.06; 0.33)	92.10	0.006	

Note: SMD, standard mean difference; CI, confidence interval; VO_2max_, maximal oxygen uptake; I^2^ = I-squared.
